# Supplementary material for: Analysis of PPARγ Signaling Activity in Psoriasis
Source: Int J Mol Sci. 2021 Aug 10;22(16):8603. doi: 10.3390/ijms22168603 (PMC8395241; doi:10.3390/ijms22168603)
Supplement: Supplementary file 1 [file ijms-22-08603-s001.zip › Supplemental materials_Analysis of PPARg signaling activity in psoriasis/Pathway models/Models images and html files/Interleukin-17 and interleukin-22 signaling in psoriasis/4683.html]

CXCL1


# Protein CXCL1

|  |  |
| --- | --- |
| URN | urn:agi-llid:2493 |
| Total Entities | 0 |
| Connectivity | 3674 |
| Name | CXCL1 |
| Description | chemokine (C-X-C motif) ligand 1 (melanoma growth stimulating activity, alpha) |
| Notes | This antimicrobial gene encodes a member of the CXC subfamily of chemokines. The encoded protein is a secreted growth factor that signals through the G-protein coupled receptor, CXC receptor 2. This protein plays a role in inflammation and as a chemoattractant for neutrophils. Aberrant expression of this protein is associated with the growth and progression of certain tumors. A naturally occurring processed form of this protein has increased chemotactic activity. Alternate splicing results in coding and non-coding variants of this gene. A pseudogene of this gene is found on chromosome 4. [provided by RefSeq, Sep 2014] |
| Primary Cell Localization | Extracellular |
| Class | Ligand |

---

|  |  |
| --- | --- |
| Pathway | CXC Chemokine Receptor Signaling |
|  | Toll-like Receptor Independent Sterile Inflammation |
|  | Proteins Involved in Atherosclerosis |
|  | Chemokines Signaling in Atherosclerosis |
|  | IL17 Signaling in Psoriasis |
|  | Proteins Involved in non-Alcoholic Fatty Liver Disease |
|  | CagA Phosphorylation Independent Signaling |
|  | Leukocyte Migration toward the Endothelial Cell in Ulcerative Colitis |
|  | Il17 Signaling Related Neutrophilia in Asthma |
|  | Neutrophil Recruitment in Sinovium in Gout |
|  | Cytokines Trigger Otitis Media |
|  | Alveolar Macrophages Dysfunction in COPD |
|  | Proteins Involved in Chronic Obstructive Pulmonary Disease |
|  | Endothelial Cell Dysfunction in Pyelonephritis |
|  | HMGB1 Expression Targets |
|  | CXCL1 Expression Targets |
|  | TAM Receptor Signaling Activation in Cancer |
|  | F2R/F2RL1 -> Expression Targets in Lymphoid System and Blood |
|  | TNF Receptors -> Expression Targets in Lymphoid System and Blood |
|  | Interleukin Receptors -> Expression Targets in Lymphoid System and Blood |
|  | TNF -> NF-kB Expression Targets |
|  | LTA Expression Targets |
|  | TLR1 -> 2/6 Expression Targets |
|  | IL1B Expression Targets |
|  | TLR4 -> NF-kB/IRF Expression Targets |
|  | IL1A Expression Targets |
|  | pprarg neg, uknown targets, ps-positive |
|  | prarg neg,ukn expres targets, ps-positive |
|  | PPARG negative targets |
|  | pparg expr target\_regulators |
|  | Neighbors of disease exacerbation |
|  | Neighbors of tumor progression |
|  | Neighbors of cancer progression |
|  | Neighbors of overall survival |
|  | Neighbors of disease severity |
|  | Neighbors of mortality |
|  | Neighbors of survival rate |
|  | Neighbors of functional recovery |
|  | Neighbors of bacterial load |
|  | Neighbors of clinical stage |
|  | Neighbors of lethality |
|  | Neighbors of tumor regression |
|  | Neighbors of disease-free survival |
|  | Neighbors of long-term survival |
|  | Neighbors of treatment outcome |
|  | Neighbors of microvessel density |
|  | Neighbors of tumor recurrence |
|  | Neighbors of tumor promotion |
|  | Neighbors of neutrophil count |
|  | Neighbors of radioresistance |
|  | Neighbors of cell density |
|  | Neighbors of shear stress |
|  | Neighbors of leukocyte count |
|  | Neighbors of vascular leakage |
|  | Neighbors of cancer recurrence |
|  | Neighbors of antimetastatic activity |
|  | Neighbors of proliferation index |
|  | Neighbors of pregnancy outcome |
|  | Neighbors of monocyte count |
|  | Neighbors of disease exacerbation |
|  | Neighbors of tumor progression |
|  | Neighbors of cancer progression |
|  | Neighbors of overall survival |
|  | Neighbors of disease severity |
|  | Neighbors of mortality |
|  | Neighbors of survival rate |
|  | Neighbors of functional recovery |
|  | Neighbors of bacterial load |
|  | Neighbors of clinical stage |
|  | Neighbors of lethality |
|  | Neighbors of tumor regression |
|  | Neighbors of disease-free survival |
|  | Neighbors of long-term survival |
|  | Neighbors of treatment outcome |
|  | Neighbors of microvessel density |
|  | Neighbors of tumor recurrence |
|  | Neighbors of tumor promotion |
|  | Neighbors of neutrophil count |
|  | Neighbors of radioresistance |
|  | Neighbors of cell density |
|  | Neighbors of shear stress |
|  | Neighbors of leukocyte count |
|  | Neighbors of vascular leakage |
|  | Neighbors of cancer recurrence |
|  | Neighbors of antimetastatic activity |
|  | Neighbors of proliferation index |
|  | Neighbors of pregnancy outcome |
|  | Neighbors of monocyte count |
|  | Neighbors of immune response |
|  | Neighbors of cell population |
|  | Neighbors of inflammatory response |
|  | Neighbors of wound healing |
|  | Neighbors of angiogenesis |
|  | Neighbors of innate immune response |
|  | Neighbors of cell function |
|  | Neighbors of cell formation |
|  | Neighbors of adaptive immune response |
|  | Neighbors of osteoclast development |
|  | Neighbors of epithelial to mesenchymal transition |
|  | Neighbors of immunity |
|  | Neighbors of cell proliferative response |
|  | Neighbors of chemotaxis |
|  | Neighbors of dendritic cell differentiation |
|  | Neighbors of cell survival |
|  | Neighbors of osteoclast differentiation |
|  | Neighbors of cell phenotype |
|  | Neighbors of T-cell proliferation |
|  | Neighbors of tissue remodeling |
|  | Neighbors of cell adhesion |
|  | Neighbors of T-cell activation |
|  | Neighbors of endothelial cell proliferation |
|  | Neighbors of fibrogenesis |
|  | Neighbors of tumor growth |
|  | Neighbors of dendritic cell development |
|  | Neighbors of cell count |
|  | Neighbors of cell infiltration |
|  | Neighbors of cell interaction |
|  | Neighbors of lumen formation |
|  | Neighbors of endothelial cell migration |
|  | Neighbors of lipid storage |
|  | Neighbors of cell damage |
|  | Neighbors of pregnancy |
|  | Neighbors of cell motility |
|  | Neighbors of monocyte migration |
|  | Neighbors of macrophage migration |
|  | Neighbors of microglial activation |
|  | Neighbors of neutrophil recruitment |
|  | Neighbors of regeneration |
|  | Neighbors of cell migration |
|  | Neighbors of cancer growth |
|  | Neighbors of stem cell proliferation |
|  | Neighbors of transendothelial migration |
|  | Neighbors of phagocytosis |
|  | Neighbors of immune system activation |
|  | Neighbors of leukocyte migration |
|  | Neighbors of leukocyte recruitment |
|  | Neighbors of osteoclast formation |
|  | Neighbors of monocyte recruitment |
|  | Neighbors of cell invasion |
|  | Neighbors of heart function |
|  | Neighbors of ossification |
|  | Neighbors of autophagy |
|  | Neighbors of monocyte adhesion |
|  | Neighbors of transcription activation |
|  | Neighbors of senescence |
|  | Neighbors of T-cell response |
|  | Neighbors of tumor immunity |
|  | Neighbors of hepatocyte proliferation |
|  | Neighbors of neuroprotection |
|  | Neighbors of stem cell differentiation |
|  | Neighbors of endothelial cell function |
|  | Neighbors of neuronal death |
|  | Neighbors of cell growth |
|  | Neighbors of ROS generation |
|  | Neighbors of epithelial cell proliferation |
|  | Neighbors of cancer cell growth |
|  | Neighbors of sensitization |
|  | Neighbors of hemato-encephalic barrier |
|  | Neighbors of cellular immune response |
|  | Neighbors of cellular senescence |
|  | Neighbors of colony formation |
|  | Neighbors of macrophage differentiation |
|  | Neighbors of cell differentiation |
|  | Neighbors of keratinocyte proliferation |
|  | Neighbors of stem cell migration |
|  | Neighbors of life span |
|  | Neighbors of cell homeostasis |
|  | Neighbors of psoriasis |
|  | Neighbors of inflammatory disease |
|  | Neighbors of rheumatoid arthritis |
|  | Neighbors of atherosclerosis |
|  | Neighbors of fibrosis |
|  | Neighbors of colitis |
|  | Neighbors of arthritis |
|  | Neighbors of injury |
|  | Neighbors of atherogenesis |
|  | Neighbors of metastasis |
|  | Neighbors of carcinogenesis |
|  | Neighbors of multiple sclerosis |
|  | Neighbors of inflammation |
|  | Neighbors of tumor microenvironment |
|  | Neighbors of pathological angiogenesis |
|  | Neighbors of obesity |
|  | Neighbors of vascular disease |
|  | Neighbors of diabetes mellitus |
|  | Neighbors of asthma |
|  | Neighbors of pneumonia |
|  | Neighbors of osteoarthritis |
|  | Neighbors of chronic obstructive pulmonary disease |
|  | Neighbors of myocardial infarction |
|  | Neighbors of leukocyte infiltration |
|  | Neighbors of reperfusion injury |
|  | Neighbors of airway inflammation |
|  | Neighbors of hepatitis |
|  | Neighbors of infection |
|  | Neighbors of vascular injury |
|  | Neighbors of renal injury |
|  | Neighbors of preeclampsia |
|  | Neighbors of macrophage infiltration |
|  | Neighbors of liver injury |
|  | Neighbors of neuroinflammation |
|  | Neighbors of sepsis |
|  | Neighbors of death |
|  | Neighbors of encephalomyelitis |
|  | Neighbors of dermatitis |
|  | Neighbors of neutrophil infiltration |
|  | Neighbors of heart disease |
|  | Neighbors of lung injury |
|  | Neighbors of hepatocellular carcinoma |
|  | Neighbors of autoimmunity |
|  | Neighbors of gastric cancer |
|  | Neighbors of atherosclerotic plaque |
|  | Neighbors of colorectal cancer |
|  | Neighbors of breast cancer |
|  | Neighbors of ischemia |
|  | Neighbors of melanoma |
|  | Neighbors of cartilage degeneration |
|  | Neighbors of neoplasm invasion |
|  | Neighbors of heart failure |
|  | Neighbors of lung disease |
|  | Neighbors of stroke |
|  | Neighbors of cancer |
|  | Neighbors of type 2 diabetes |
|  | Neighbors of neoplasm |
|  | Neighbors of neointima |
|  | Neighbors of diet-induced obesity |
|  | Neighbors of myocardial fibrosis |
|  | Neighbors of synovitis |
|  | Neighbors of coronary artery disease |
|  | Neighbors of rupture |
|  | Neighbors of endothelial cell dysfunction |
|  | Neighbors of prostate cancer |
|  | Neighbors of hypertrophy |
|  | Neighbors of osteolysis |
|  | Neighbors of ovarian cancer |
|  | Neighbors of pancreatic cancer |
|  | Neighbors of hypertension |
|  | Neighbors of allograft rejection |
|  | Neighbors of epithelial cell |
|  | Neighbors of keratinocyte |
|  | Neighbors of vascular smooth muscle cell |
|  | Neighbors of umbilical vein endothelial cell |
|  | Neighbors of monocyte |
|  | Neighbors of endometrial stromal cell |
|  | Neighbors of alveolar macrophage |
|  | Neighbors of synoviocyte |
|  | Neighbors of macrophage |
|  | Neighbors of adipocyte |
|  | Neighbors of activated monocyte |
|  | Neighbors of fibroblast |
|  | Neighbors of airway epithelial cell |
|  | Neighbors of PBMC |
|  | Neighbors of stromal cell |
|  | Neighbors of airway smooth muscle cell |
|  | Neighbors of osteoblast |
|  | Neighbors of pneumocyte |
|  | Neighbors of microglia |
|  | Neighbors of trophoblast |
|  | Neighbors of intestine epithelium cell |
|  | Neighbors of hepatic stellate cell |
|  | Neighbors of hepatocyte |
|  | Neighbors of kidney mesangial cell |
|  | Neighbors of endothelial cell |
|  | Neighbors of dendritic cell |
|  | Neighbors of Kuppfer cell |
|  | Neighbors of proadipocyte |
|  | Neighbors of kidney tubule cell |
|  | Neighbors of inflammatory cell |
|  | Neighbors of bone marrow macrophage |
|  | Neighbors of peritoneal macrophage |
|  | Neighbors of osteoclast |
|  | Neighbors of neutrophil |
|  | Neighbors of smooth muscle myocyte |
|  | Neighbors of astrocyte |
|  | Neighbors of bone marrow derived dendritic cell |
|  | Neighbors of lung fibroblast |
|  | Neighbors of activated T-cell |
|  | Neighbors of mesothelial cell |
|  | Neighbors of mononuclear phagocyte |
|  | Neighbors of capillary endothelial cell |
|  | Neighbors of immunocompetent cell |
|  | Neighbors of granulocyte |
|  | Neighbors of cardiac myocyte |
|  | Neighbors of cancer-associated fibroblast |
|  | Neighbors of myeloid cell |
|  | Neighbors of mesenchymal stroma cell |
|  | Neighbors of stem cell |
|  | Neighbors of tumor infiltrating macrophage |
|  | Neighbors of B-cell |
|  | Neighbors of granulosa cell |
|  | Neighbors of leukocyte |
|  | Neighbors of chondrocyte |
|  | Neighbors of insulin-secreting cell |
|  | Neighbors of mesenchymal cell |
|  | Neighbors of T-cell |
|  | Neighbors of myofibroblast |
|  | Neighbors of pancreatic stellate cell |
|  | Neighbors of mast cell |
|  | Neighbors of spleen cell |
|  | Neighbors of brain capillary endothelial cell |
|  | Neighbors of neuroglia |
|  | Neighbors of hematopoietic cell |
|  | Neighbors of podocyte |
|  | Neighbors of mammary epithelial cell |
|  | Neighbors of pericyte |
|  | Neighbors of epidermis cell |
|  | Neighbors of bone marrow derived mast cell |
|  | Neighbors of phagocyte |
|  | Neighbors of lymphocyte |
|  | Neighbors of thymic epithelial cell |
|  | Neighbors of brain cell |
|  | Neighbors of M2 macrophage |
|  | Neighbors of peritoneum cell |
|  | Neighbors of lymphoid cell |
|  | Neighbors of retina cell |
|  | Neighbors of skin cell |
|  | Neighbors of lung |
|  | Neighbors of liver |
|  | Neighbors of kidney |
|  | Neighbors of skin |
|  | Neighbors of lymph node |
|  | Neighbors of colon |
|  | Neighbors of airway |
|  | Neighbors of blood vessel |
|  | Neighbors of heart |
|  | Neighbors of aorta |
|  | Neighbors of microvessel |
|  | Neighbors of placenta |
|  | Neighbors of synovial membrane |
|  | Neighbors of brain |
|  | Neighbors of stomach |
|  | Neighbors of islets of Langerhans |
|  | Neighbors of joint |
|  | Neighbors of prostate |
|  | Neighbors of spleen |
|  | Neighbors of allantois |
|  | Neighbors of hippocampus |
|  | Neighbors of cerebral cortex |
|  | Neighbors of draining lymph node |
|  | Neighbors of cervical lymph node group |
|  | Neighbors of amniotic cavity |
|  | Neighbors of NF-kB family |
|  | Neighbors of oxidized LDL |
|  | Neighbors of JNK |
|  | Neighbors of IL1 family |
|  | Neighbors of Jun/Fos |
|  | Neighbors of NFKBI |
|  | Neighbors of PKC |
|  | Neighbors of PI3K |
|  | Neighbors of mitogen-activated protein kinase |
|  | Neighbors of IFNAR ligand |
|  | Neighbors of transforming growth factor |
|  | Neighbors of TLR |
|  | Neighbors of cytokine |
|  | Neighbors of interferon |
|  | Neighbors of interleukin |
|  | Neighbors of PDGF |
|  | Neighbors of Ras GTPase |
|  | Neighbors of PKA |
|  | Neighbors of ovalbumin |
|  | Neighbors of NF-AT family |
|  | Neighbors of beta adrenoceptor |
|  | Neighbors of IKBK |
|  | Neighbors of protein tyrosine kinase |
|  | Neighbors of NAD(P)H oxidase |
|  | Neighbors of IgG |
|  | Neighbors of SMAD subfamily |
|  | Neighbors of LXR |
|  | Neighbors of HDL |
|  | Neighbors of inflammatory cytokine |
|  | Neighbors of IL23 |
|  | Neighbors of histone deacetylase |
|  | Neighbors of IL1R |
|  | Neighbors of lectin |
|  | Neighbors of fibrinogen |
|  | Neighbors of mitogen-activated protein kinase kinase |
|  | Neighbors of interleukin-35 |
|  | Neighbors of endothelin |
|  | Neighbors of proteasome endopeptidase complex |
|  | Neighbors of histone H3 |
|  | Neighbors of PP2A |
|  | Neighbors of phospholipase |
|  | Neighbors of AHR subfamily |
|  | Neighbors of ITG |
|  | Neighbors of histone |
|  | Neighbors of Rho kinase |
|  | Neighbors of pancreatic elastase |
|  | Neighbors of phosphoinositide phospholipase C |
|  | PPARG negative regulators and targets |
|  | Model of PPARG related pathways in psoriasis (short version) |
|  | Overview. Endocrine and metabolic diseases (chapter 4) |
|  | 2\_Interleukin-17 and interleukin-22 signaling in psoriasis |
|  | 2\_The role of endothelial cells in pyelonephritis |
|  | 3\_Role of alveolar macrophages in pulmonary tissue destruction in COPD |
|  | 3\_Leukocyte migration toward the endothelial cells in the intestine microvasculature |
|  | drug-target moltransport |
|  | drug-target molsynthesis |
|  | Interleukin-17 and interleukin-22 signaling in psoriasis |

---

|  |  |
| --- | --- |
| Group | Chemokines, Growth Factors and Receptors Involved in Cancer Cells Chemotaxis |

---

|  |  |
| --- | --- |
| MedScan ID | 2493 |

---

|  |  |
| --- | --- |
| LocusLink ID | 2493 |
|  | 2919 |
|  | 330122 |
|  | 171551 |

---

|  |  |
| --- | --- |
| Alias | chemokine (C-X-C motif) ligand I |
|  | melanoma growth-stimulating activity |
|  | melanoma growth-stimulation activity |
|  | MGSA alpha |
|  | GRO1 oncogene |
|  | small inducible cytokine B1 |
|  | melanoma growth stimulatory activity factor alpha |
|  | MGSA/GRO |
|  | Cxcl I |
|  | Growth-regulated oncogene alpha |
|  | GRO-alpha(1-73) |
|  | GROA |
|  | GRO alpha |
|  | Fsp |
|  | SCYB1 |
|  | CXCL1 chemokine |
|  | cytokine induced neutrophil chemokine CINC-1 |
|  | NAP III |
|  | chemokine (C-X-C motif) ligand 1 |
|  | Neutrophil-activating protein III |
|  | melanoma growth-stimulatory activity |
|  | CINC-1 |
|  | MGSA-a |
|  | GRO1 |
|  | CXC chemokine ligand 1 |
|  | Neutrophil-activating protein 3 |
|  | GRO1 oncogene (melanoma growth-stimulating activity) |
|  | SCYB I |
|  | melanoma growth-stimulatory activity factor |
|  | NAP-3 |
|  | MGSA |
|  | chemokine (C-X-C motif) ligand 1 (melanoma growth stimulating activity, alpha) |
|  | chemokine CXC ligand 1 |
|  | N51 protein |
|  | C-X-C motif chemokine ligand 1 |
|  | melanoma growth-stimulatory activity factors |
|  | melanoma growth stimulating activity, alpha |
|  | cytokine-induced neutrophil chemoattractant I |
|  | melanoma growth stimulatory activity alpha |
|  | CXCL1 |
|  | Growth regulated protein precursor alpha |
|  | CXC ligand 1 |
|  | cytokine-induced neutrophil chemoattractant 1 |
|  | fibroblast secretory protein |
|  | chemokine CXCL1 |
|  | GRO I |
|  | GRO\_HUMAN |
|  | GRO1 oncogene (melanoma growth stimulating activity, alpha) |
|  | C-X-C motif chemokine 1 |
|  | growth-regulated alpha protein |
|  | Dcip1 |
|  | Gm1960 |
|  | C-X-C motif chemokine 3 |
|  | dendritic cell inflammatory protein 1 |
|  | Cinc2 |
|  | Cinc-2 |
|  | MIP2-alpha/beta |
|  | macrophage inflammatory protein 2-alpha/beta |
|  | cytokine-induced neutrophil chemoattractant 2 |
|  | cytokine-induced neutrophil chemoattractant-2 |
|  | NAP IIIs |
|  | small inducible cytokine B subfamily |
|  | gro |

---

|  |  |
| --- | --- |
| GO ID | 0008009 |
|  | 0008047 |
|  | 0008083 |
|  | 0005102 |
|  | 0007186 |
|  | 0030036 |
|  | 0061844 |
|  | 0071222 |
|  | 0070098 |
|  | 0006935 |
|  | 0019221 |
|  | 0006955 |
|  | 0006954 |
|  | 0035556 |
|  | 0030595 |
|  | 0008285 |
|  | 0007399 |
|  | 0030593 |
|  | 0043312 |
|  | 0043085 |
|  | 0007165 |
|  | 0005576 |
|  | 0005615 |
|  | 0035580 |
|  | 1904724 |
|  | 0045236 |
|  | 0060326 |
|  | 0008283 |
|  | 0090023 |
|  | 0032496 |
|  | 0005622 |
|  | 0002690 |
|  | 0005125 |
|  | 0007204 |

---

|  |  |
| --- | --- |
| KEGG ID | hsa:2919 |
|  | mmu:330122 |
|  | rno:171551 |

---

|  |  |
| --- | --- |
| Organism | Homo sapiens {Organism urn:agi-taxid:9606} |
|  | Homo sapiens |
|  | Mus musculus |
|  | Rattus norvegicus |

---

|  |  |
| --- | --- |
| Mouse chromosome position | 5 |

---

|  |  |
| --- | --- |
| OMIM ID | 155730 |

---

|  |  |
| --- | --- |
| Rat chromosome position | 14p22 |

---

|  |  |
| --- | --- |
| Hugo ID | 4602 |
|  | HGNC:4602 |

---

|  |  |
| --- | --- |
| Human chromosome position | 4q13.3 |
|  | 4q21 |

---

|  |  |
| --- | --- |
| Swiss-Prot Accession | P09341 |
|  | P09341.1 |
|  | Q9UCR7 |
|  | Q6W5C0 |
|  | Q10746 |
|  | Q10747 |
|  | Q5CZ55 |
|  | Q9EP62 |
|  | Q3UNK9 |

---

|  |  |
| --- | --- |
| PIR ID | S13669 |
|  | S46198 |

---

|  |  |
| --- | --- |
| GenBank ID | NC\_000004 |
|  | NR\_046035 |
|  | NM\_001511 |
|  | NP\_001502 |
|  | AC092438 |
|  | CH471057 |
|  | EAX05693 |
|  | HI519534 |
|  | CBX54348 |
|  | JA514411 |
|  | CCD62019 |
|  | M65005 |
|  | AAP13103 |
|  | U03018 |
|  | AAA03452 |
|  | X54489 |
|  | CAA38361 |
|  | BC011976 |
|  | AAH11976 |
|  | BG121608 |
|  | BM554448 |
|  | BM996663 |
|  | BQ000742 |
|  | BT006880 |
|  | AAP35526 |
|  | CB112830 |
|  | J03561 |
|  | AAA35933 |
|  | X12510 |
|  | CAA31027 |
|  | P09341 |
|  | NC\_018915 |
|  | AMYH02009472 |
|  | DQ893591 |
|  | ABM84517 |
|  | DQ894941 |
|  | ABM85867 |
|  | AC\_000136 |
|  | ABBA01022993 |
|  | NC\_000071 |
|  | NM\_203320 |
|  | NP\_976065 |
|  | NT\_109599 |
|  | AC\_000027 |
|  | AC105995 |
|  | AC113262 |
|  | AY311404 |
|  | AAQ86937 |
|  | CH466617 |
|  | EDL05303 |
|  | AK144158 |
|  | BAE25738 |
|  | AK150314 |
|  | BAE29460 |
|  | AK154334 |
|  | BAE32520 |
|  | AK155702 |
|  | AY311403 |
|  | AAQ86936 |
|  | BC117014 |
|  | AAI17015 |
|  | BC117016 |
|  | AAI17017 |
|  | Q6W5C0 |
|  | NC\_005113 |
|  | NM\_138522 |
|  | NP\_612531 |
|  | AC\_000082 |
|  | CH474060 |
|  | EDL88570 |
|  | EDL88571 |
|  | EDL88572 |
|  | EDL88573 |
|  | D87926 |
|  | BAB12279 |
|  | BAB12336 |
|  | D21095 |
|  | BAA04657 |
|  | D87927 |
|  | BAB12280 |
|  | Q10746 |

---

|  |  |
| --- | --- |
| Swiss-Prot ID | GROA\_HUMAN |
|  | CXCL3\_RAT |
|  | CXCL3\_MOUSE |

---

|  |  |
| --- | --- |
| Cell Localization | Secreted |
|  | Extracellular |

---

|  |  |
| --- | --- |
| Ensembl ID | ENSG00000163739 |
|  | ENSP00000379110.3 |
|  | ENST00000395761.3 |
|  | ENSP00000379110 |
|  | ENST00000395761 |

---

|  |  |
| --- | --- |
| MGI ID | 3037818 |

---

|  |  |
| --- | --- |
| RGD ID | 621812 |

---

|  |  |
| --- | --- |
| Unigene ID | Hs.789 |
|  | Hs.708652 |
|  | Mm.244289 |
|  | Rn.10525 |

---

|  |  |
| --- | --- |
| Homologene ID | 117693 |

---

|  |  |
| --- | --- |
| Shape | Rhomb |

---

|  |  |
| --- | --- |
| IPI ID | IPI00366205 |
|  | IPI00778837 |
|  | IPI00013874 |
|  | IPI00356181 |

---
